# Risk factors of periodontal disease: Latin America and the Caribbean Consensus 2024

**DOI:** 10.1590/1807-3107bor-2024.vol38.0118

**Published:** 2024-11-22

**Authors:** Claudio Mendes PANNUTI, Marco Antonio ALARCÓN, Gloria Marcela RAMÍREZ LEMUS, Paula YUNES FRAGOSO, Belen Stephanie RETAMAL-VALDES, Marco CORNEJO-OVALLE, Poliana Mendes DUARTE, Fabio Renato Manzolli LEITE, Xiomara GIMENEZ

**Affiliations:** (a) Universidade de São Paulo – USP, School of Dentistry, Department of Stomatology, São Paulo, SP, Brazil.; (b) Universidad Peruana Cayetano Heredia, Academic Department of Clinical Stomatology, Lima, Perú.; (c) Pontificia Universidad Javeriana, Bogotá, Colombia; (d) Universidad Iberoamericana, Biomaterials and Dentistry Research Center, Research and Innovation Department, Santo Domingo, Dominican Republic.; (e) RS Master Saúde, Department of Periodontology, Arujá, Brazil.; (f) Universidad de Chile, Facultad de Odontología, Instituto de Investigación, Santiago, Chile.; (g) University of Florida, College of Dentistry, Department of Periodontology, Gainesville, FL, USA.; (h) National Dental Research Institute Singapore –NDRIS, National Dental Centre Singapore, Singapore.; (i) Universidad Central de Venezuela. Caracas, Venezuela.

**Keywords:** Periodontitis, Risk Factors, Smoking, Diabetes Mellitus

## Abstract

There is scarcity of information on the determinants of periodontitis in Latin America and Caribbean countries. We conducted a comprehensive review of studies examining the association of smoking and diabetes with periodontitis outcomes in this region. We searched for population-based, cross-sectional and prospective cohort studies from Latin America and the Caribbean region that reported on the association between smoking or diabetes and periodontitis. Databases were searched up to October 2023 by two reviewers. Subsequently, two authors independently conducted a rigorous data extraction process, focusing on study characteristics, the definition of exposures, and periodontitis outcomes, including measures of association and main findings. The results revealed a significant association between smoking and periodontitis, with a stronger effect observed in heavy smokers. Conversely, while some studies observed a higher prevalence of periodontitis among diabetic individuals, the association between diabetes and periodontitis was not significant after adjusting for confounding factors. These findings underscore a significant research gap in population-based studies on the effect of smoking and diabetes on periodontitis within Latin American and Caribbean countries, particularly when it comes to cohort studies. Addressing these gaps is crucial for a deeper understanding of these associations, which could lead to more effective prevention and treatment strategies in the region.

## Introduction

Periodontitis poses a public health problem due to its high prevalence, chronic nature, impact on quality of life, and role in health disparities.^1^ Moreover, the economic burden associated with its treatment costs places strains on healthcare systems.^2^

As with many non-communicable diseases, such as cancer, type 2 diabetes mellitus and cardiovascular diseases, the development and progression of periodontitis are influenced by a combination of genetic, environmental, and behavioral factors.^3^

Some exposures that have been associated with periodontitis include age, socioeconomic status, stress, obesity and genetic predisposition.^4-6^ Evidence robustly identifies smoking and type 2 diabetes as well-established causes of periodontitis^7-9^ that play pivotal roles not only in disease pathogenesis but also in its prevention and treatment response.^10,11^

This paper reviews the studies investigating the association of smoking and diabetes with periodontitis outcomes in Latin America and Caribbean countries. Information synthesis in areas such as these is crucial for several reasons. Firstly, Latin American populations may encounter unique genetic, environmental, and lifestyle factors influencing these associations, emphasizing the need to tailored public health interventions and clinical management. Secondly, understanding the findings can help address health inequities, facilitate targeted awareness and prevention programs, and guide the development of effective public health interventions. Furthermore, it can foster interdisciplinary collaboration among health professionals and public health experts, ultimately enhancing oral and systemic health outcomes in this region.

## Methods

### Inclusion and exclusion criteria

Population-based, cross-sectional and prospective cohort studies conducted in the countries of Latin America and the Caribbean were targeted. Only those presenting data on smoking and/or diabetes as the exposure, with periodontitis as the outcome, were included. Those assessing smoking or diabetes but lacking reports of associations with periodontitis were excluded.

### Search strategy

MEDLINE-PubMed, SCOPUS, and EMBASE databases were searched up to October 2023. The search strategy can be found on the Open Science Framework webpage (https://osf.io/wt5bv/?view_only=d3fdff9e108e43c6ad57227a195f17ba). Two authors (PM and GR) independently screened the retrieved articles. Studies that met the inclusion criteria and those with insufficient information in titles and abstracts were submitted to full manuscript evaluation. Subsequently, the studies selected were submitted to data extraction.

### Data extraction

Two reviewers (MAAP and BRV) independently extracted the following data:

Publication details: authors and publication year.

Characteristics of the study: country of data collection, study design, sample characteristics, sample size calculation, potential confounders and other variables, methods used to assess diabetes and/or smoking (exposures), and periodontitis (outcome).

Results: measure of association between the exposure and periodontitis, and the main findings.

After data extraction, a descriptive analysis of the articles was performed.

## Results

For the association between smoking and periodontitis, 230 publications in PUBMED, 225 in Embase, and 258 in Scopus were found (total: 713 hits with 211 duplicates, resulting in 502 publications to read title and abstract). For the relationship between diabetes and periodontitis, 114 publications in PUBMED, 163 in Embase, and 184 in Scopus were retrieved (Total: 461 hits with 143 duplicates, resulting in 318 publications to analyze titles and abstracts). After full-text reading, data were extracted from 11 studies.

### Smoking

A total of 11 studies on the association between smoking and periodontitis were included (Table 1). Eight studies were conducted in Brazil,^12-19^ two in Chile^20,21^ and one in Colombia.^22^ The majority of the studies were cross-sectional, except for two prospective population-based cohorts: one with a 5-year follow-up^18^ and another with individuals born in 1982.^19^

**Table 1:** Descriptive analysis of the articles related to smoking and periodontitis.

Authors (year)	Country	Study design	Population and sample	Age range	Prior sample size estimation	Exposure definition (DM)	Outcome definition	Other variables	Association of periodontitis with smoking. Effect size [95%IC]	Main findings
López et al. (2001)^20^	Chile	Cross-sectional	9,203 students from 98 high schools in the province of Santiago	12–21	Yes	Number of packs smoked, defined as “the number of cigarettes smoked daily *365* duration of smoking in years/20.”	Occurrence of ≥ 2 teeth with interproximal CAL ≥1 mm, or ≥ 1 tooth with interproximal CAL ≥ 3 mm	Age, gender, tooth brushing frequency, last visit to the dentist, governmental support, Diabetes	OR - Presence of ≥ 2 teeth with CAL ≥ 1mm:	The study failed to demonstrate a strong and statistically significant association between smoking and CAL
						Exposure was categorized as:			All sites	
						1 to 250,			1-250: 0.90 [0.78–1.03]	
						251 to 500,			251-500: 1.00 [0.83–1.20]	
						> 500 packs.			> 500: 1.16 [0.93–1.46]	
	
									Interproximal only	
									1-250: 0.94 [0.82–1.07]	
									251-500: 1.00 [0.83–1.21]	
									> 500: 1.15 [0.93–1.43]	
	
									OR - Presence of ≥ 1 teeth with CAL ≥ 3mm:	
									All sites	
									1-250: 1.02 [0.74–1.41]	
									251-500: 0.96 [0.64–1.44]	
									> 500: 1.14 [0.75–1.74]	
	
									Interproximal only	
									1-250: 0.98 [0.71–1.36]	
									251-500: 0.98 [0.63–1.53]	
									> 500: 1.10 [0.68–1.74]	
Susin et al. (2004)^12^	Brazil	Cross-sectional	843 subjects >30 years living in the metropolitan area of Porto Alegre	30–103	Yes	Number of packs consumed in a life-time: number of cigarettes smoked per day multiplied by the number of years of habit, and divided by 20 (one pack):	Percentage of teeth with:	Age, gender, race, socioeconomic status, dental visits, Diabetes	CAL (RRR)	Aging and moderate to heavy cigarette smoking significantly increased the risk for moderate and severe CAL
						Non-smokers, light (1 to 2,734 packs), moderate (2,735 to 7,300 packs) and heavy smokers (≥7,300 packs).	Severe CAL: CAL ≥ 5mm in > 50% of teeth			
									Non-smoker (reference)	
							Moderate CAL: CAL ≥ 5mm in 15% to 50% of teeth			
									Moderate CAL	
							Slight or no CAL: below moderate category		Light: 1.1 [0.7–1.9]	
									Moderate: 2.1 [1.4–3.2]*	
									Heavy: 3.0 [1.6–5.8]*	
		
									Severe CAL	
									Light: 1.4 [0.6–3.2]	
									Moderate: 3.4 [2.6–4.4]*	
									Heavy: 8.2 [5.5–12.2]*	
Susin; Albandar (2005)^13^	Brazil	Cross-sectional	612 youngsters	14–29	Yes	Non-smokers (<1 cigarette packs in a lifetime)	AgP depending on age:	Socioeconomic status	AgP (OR):	Smoking was a significant risk indicator for AgP in this young population.
			living in the metropolitan area of Porto Alegre			Light (1 to 912 packs)	Aged 14-19: >4 teeth with CAL ≥4 mm.	Supragingival calculus	None (reference)	
						Moderate/ heavy smokers (>912 packs)	Aged 20-29: >4 teeth with CAL ≥5 mm.		Light: 0.6 [0.1–2.4]	
									Moderate/heavy: 3.1 [1.2–8.3]*	
Moimaz et al. (2009)^14^	Brazil	Cross-sectional	165 individuals >30 years living in rural Araçatuba	35–66	NR	Number of packs smoked: number of cigarettes smoked daily multiplied by the number of days of habit and divided by 20 (one pack).	Presence of periodontal pockets, defined as having ≥1 periodontal pocket of ≥ 4 mm around the index teeth (CPI scores: 3 and 4)	NR	Presence of periodontal pockets (OR)	Smoking was strongly associated with periodontitis. There was a relationship with dose and duration of smoking.
						Former smoker: who had smoked in the past but do not smoke any more.			Non-smokers: reference	
						Never- smokers			Current smokers: 11.18 [4.69–26.62]*	
						Light (1 to 2,734 packs)			Former smokers: 9.24 [3.29–25.96]*	
						Moderate (2,735 to 7,300 packs)				
						Heavy smokers (≥7,300 packs)				
Gamonal et al. (2010)^21^	Chile	Cross-sectional	1561 adults. Clinical evaluations in dental public primary care health centers	Two groups:	Yes	Never-smokers or Smokers (current or former smokers).	Prevalence of CAL, defined as the percentage of participants with ≥1 site with the condition. Extent was defined as the percentage of teeth displaying the condition.	Age, sex, Education, monthly income,	CAL (OR):	Age (65 to 74 years), sex (male), low education level (≤12 years of education), and smoking were risk indicators for CAL >6 mm in ≥1 site
				35–44				Diabetes	≥3 mm 1.4 [0.9–2.3]	
									≥4 mm 1.3 [1.0–1.8]*	
				65–74					≥5 mm 1.3 [1.0–1.6]*	
									≥6 mm 1.3 [1.0–1.7]*	
Silva et al. (2010)^15^	Brazil	Cross-sectional	300 individuals with diabetes from	30–86	Yes	NR	Prevalence of periodontitis defined as CAL ≥ 3 mm in ≥ 2 non-adjacent teeth or CAL ≥ 5 mm in 30% of the teeth (EFP; Tonetti & Claffey, 2005).	Gender, age, income, schooling, marital status, missing teeth, dental care, diabetes	CAL (PR)	Multiple determinants, among them smoking, are associated to the prevalence of periodontitis among patients with diabetes
			Public health facilities in Belo Horizonte						non-smokers: Reference	
									Tobacco users: 1.71 [1.10–2.65]*	
Frias et al. (2011)^16^	Brazil	Cross-sectional	263 subjects living in Guarulhos	35–44	Yes	NR	Two outcomes: Bleeding (CPI = 1) and dental calculus (CPI = 2), and presence of moderate (CPI=3) or deep (CPI=4) periodontal pockets	Sex, education and access to dental care	Periodontal pockets (PR)	Prevalence of gingival bleeding and dental calculus were significantly associated to male sex (PR=1.12), smoking (PR=1.11), school level < eight years of study (PR=1.14), & no dental care for >2 years (PR=1.19).

									Bleeding & dental calculus	
									1.11 [1.02–1.21]*	

									Periodontal pockets	
									1.71 [1.07–2.73]*	
Susin al. (2011)^17^	Brazil	Cross-sectional	612 individuals living in the metropolitan area of Porto Alegre	14–29	Yes	Non-smokers (<1 pack of cigarettes in a lifetime)	Chronic periodontitis defined as CAL ≥3mm affecting the interproximal sites of >2 teeth.	Age, supragingival calculus,	Chronic Periodontitis	Age, socioeconomic status, smoking and supragingival calculus were significantly associated with chronic periodontitis.
						Light (1–499 packs)		Dental visits	CAL (OR)	
						Moderate (500–1499 packs)		Supragingival calculus		
						Heavy smokers (≥1500 packs).			Non-smokers = reference	
									Light/moderate: 0.9 [0.6–1.3]	
									Heavy: 1.7 [1.1–2.7]*	
Haas et al. (2014)^18^	Brazil	Prospective population-based cohort of 5 years of follow-up	653 individuals from the Metropolitan area of Porto Alegre	14	Yes	Packyears of smoking calculated by multiplying the number of packs consumed per day by the number of years of habit. To facilitate the interpretation of the results, estimates of lifetime smoking were divided by 10 so that risk estimates reflect changes in risk for 10 packyears of smoking.	CAL progression at a given site, calculated by subtracting the baseline CAL from that of the 5-year follow-up examination. Two case definitions were used with CAL progression cases, defined as individuals having proximal CAL progression ≥3mm in ≥2 and ≥4 teeth over the 5 years of follow-up	Age, gender, marital status, skin color, education, socioeconomic status, interproximal cleaning, dental care, diabetes	CAL progression ≥3mm in ≥2 teeth (RR)	Age, gender, education and smoking were independent risk factors for CAL progression in an urban population from South Brazil.
						Smoking exposure			0 = reference	
						0 packyears			1-14: 1.12 [0.91–1.40]	
						1–14 packyears			>15: 1.39 [1.10–1.76]*	
						≥15 packyears				
									CAL progression ≥3mm in ≥4 teeth (RR)	
									0 = reference	
									1-14: 1.22 [0.88–1.71]	
									>15: 1.54 [1.06–2.24]*	
Serrano; Suárez (2019)^22^	Colombia	Cross-sectional	9821 adults from a national sample of Colombian adults, held by the Colombian Health Ministry, living in urban and rural areas	18–79	Yes	Presence of smoking behavior was analyzed by a self-reported questionnaire, categorized as: current smokers,	Periodontitis according to two classification systems: 1) AAP-CDC (Page, et al., 2012): mild, moderate and severe periodontitis. Severe periodontitis cases: ≥ 2 interproximal sites with CAL ≥ 6 mm, and ≥ 1 site with PD ≥ 5 mm. 2) EFP periodontitis case definition (Tonetti and Claffey, 2005).	Age, gender, Living area (rural / urban), health insurance system, income, Toothbrushing,	Periodontitis (CDC-AAP) (OR)	Prevalence of severe periodontitis was significantly associated with age, gender, income, smoking behavior,
						occasional smokers, former smokers or non-smokers. Definitions were not reported.(NR)		Dental floss use,	Non-smoker: reference	and diabetic status.
								Reason for dental visits,	Current smoker: 1.09 [0.9–1.3]	
								Diabetes	Former smoker: 1.28 [1.2–1.4]*	
			
									Periodontitis (EFP) (OR)	
									Non-smoker: reference	
									Current smoker: 1.57 [1.2–1.7]*	
									Former smoker: 1.14 [1.0–1.2]	
Schuch et al. (2019)^19^	Brazil	Prospective population-based birth cohort	539 individuals from 3 maternity hospitals in Pelotas city.	31	Yes	Smoking status and dental hygiene at age 24 were the mediators between socioeconomic position and periodontitis. Smoking status was dichotomized as current or former smoker versus non-smoker.	Periodontitis according to CDC and AAP:	Sex, socioeconomic position, income,	Periodontitis (RR)	An association was observed between the more severe level of periodontitis and being a smoker at age 24, as well as having undertaken less than 12 years of study
			All 5,914 children born in 1982 were invited to participate.				Mild: ≥ 2 interproximal sites with CAL ≥3 mm, and ≥2 interproximal sites with PD ≥4 mm (not on the same tooth) or one site with PD ≥5 mm.	Dental calculus	CAL	
							Moderate: ≥2 interproximal sites with CAL ≥4 mm (not on the same tooth), or ≥2 interproximal sites with PD ≥5 mm (not on the same tooth).	Education	Crude RR mild periodontitis	
							Severe: ≥2 interproximal sites with CAL ≥6 mm (not on the same tooth) and ≥1 interproximal site with PD ≥5 mm.		Non-smoker: reference	
									Smoker at age 24: 1.2 [0.7–2.0]	
									
									Crude RR Moderate to Severe periodontitis	
									Non-smoker: reference	
									Smoker at age 24: 1.6 [0.9–2.8]	

* Statistically significant. AgP: aggressive periodontitis, NR: not reported; CAL: clinical attachment loss; PD: probing depth; OR: odds ratio; RRR: relative risk ratio; PR: prevalence ratio; RR: risk ratio; CPI: community periodontal index; CDC: Center for Disease Control and Prevention; AAP: American Academy of Periodontology; EFP: European Federation of Periodontology.

The sample size ranged from 165^14^ to 9,821 individuals,^22^ with ages ranging from 12^20^ to 103 years.^12^ Sample size estimation was calculated a priori in 10 of the 11 studies, apart from one study.^14^

In six studies, the definition of exposure was based on the total number of packs smoked.^12-14,17,18,20^ Two investigations categorized subjects as either smokers (current or former smokers) or never-smokers,^19,21^and the remaining three studies did not report their definition of smoking status.

Clinical attachment loss (CAL) was the main clinical outcome used to define the periodontal status in nine of the studies included. Another study used the presence of periodontal pockets as its outcome^14^ and one defined periodontitis based on the Community Periodontal Index (CPI) .^16^ Age, sex, diabetes, dental consultation attendance, education, and other socioeconomic factors were the main covariables evaluated in the studies.

A significant positive association between smoking and periodontitis was observed in the majority of the studies, with a stronger association observed among heavy smokers. After adjusting for confounding factors, the association vanished in one study using an adolescent population, probably due to the reduced sample size and more reduced exposure to tobacco along the life- course.^20^

### Diabetes

Six studies on the association between diabetes and periodontitis were included in the present review (Table 2). Three studies were developed in Brazil,^12,15,18^ two in Chile^20,21^ and one in Colombia.^22^ Five studies were cross-sectional^12,15,20,21,22^ and one was a prospective population-based cohort with a 5-year follow-up.^18^ The sample ranged from 300^15^ to 9,821 individuals^22^ aged from 12 to 103 years. Sample size estimation was performed before conducting all six studies. One study defined diabetes based on medical diagnosis,^15^ three studies used the patient’s self-report of diabetes^18,21,22^ while two articles did not report how the diabetic status was determined.^12,20^ CAL was the main clinical outcome used to define the periodontal status in all included studies. Age, sex, smoking status, education and socioeconomic factors were the other main variables evaluated in the studies. Significant positive statistical associations between diabetes and periodontitis were observed in the majority of studies,^12,15,18,21,22^ except for one.^20^ However, the association between diabetes and periodontitis was no longer significant in some studies when confounding factors were considered.^12,18,21^ A potential explanation could be the study participants’ lack of awareness of their current blood glucose level, introducing bias into the analyses by including people with high glucose levels in the ‘health’ category and also by placing people who live with diabetes but maintain adequate glucose levels in the ‘diabetes’ category.

**Table 2:** Descriptive analysis of the studies reporting the association between diabetes and periodontitis.

Authors	Country	Study design	Population and sample	Age range	Prior sample size estimation	Diabetes definition	Outcome definition	Other variables	Association of periodontitis with smoking. Effect size [95%IC]	Main findings
López et al. (2001)^20^	Chile	Cross-sectional	9,203 students from 98 high schools in the province of Santiago	12–21	Yes	NR	Occurrence of ≥ 2 teeth with interproximal CAL ≥1 mm, or ≥ 1 tooth with interproximal CAL ≥ 3 mm	Age,Author gender, tooth brushing frequency, last visit to the dentist, governmental support, smoking	OR - Presence of ≥ 2 teeth with CAL ≥ 1mm:	Diabetic status was not associated with CAL after adjusting for other variables.
									All sites	
									1.27 [95%IC: 0.74–2.19]	
									Interproximal sites	
									1.68 [95%IC: 0.98–2.86]	

									OR - Presence of ≥ 1 teeth with CAL ≥ 3mm:	
									All sites	
									1.87 [95%IC: =0.76–4.61]	
									Interproximal sites	
									1.30 [95%IC: 0.41–4.06]	
Susin et al. (2004)^12^	Brazil	Cross-sectional	843 subjects >30 years living in the metropolitan Porto Alegre area	30–103	Yes	NR	Percentage of teeth with:	Age, gender, race, socioeconomic status, dental visits, smoking	CAL (RRR)	Diabetic status was not associated with CAL after adjusting for other variables.
							Severe CAL: CAL ≥ 5mm in > 50% of teeth		Significant higher percentage of teeth with CAL ≥ 3mm and ≥ 5 mm in patients with DM.	
	
							Moderate CAL: CAL ≥ 5mm in 15% to 50% of teeth		RRR - Moderate CAL:	
									Non-diabetic: Reference	
							Slight or no CAL: below moderate category		Diabetic: 1.7 [0.8–3.5]	
	
									RRR - Severe CAL:	
									Non-diabetic: Reference	
									Diabetic: 3.3 [1.1–10.6]*	
Silva et al. (2010)^15^	Brazil	Cross-sectional	300 individuals with diabetes from	30–86	Yes	medical diagnosis	Prevalence of periodontitis defined as CAL ≥ 3 mm in ≥ 2 non-adjacent teeth or CAL ≥ 5 mm in 30% of the teeth (EFP; Tonetti & Claffey, 2005).	Gender, age, income, schooling, marital status, missing teeth, dental care, smoking	CAL (PR)	More than 8 years duration of DM was associated with periodontitis.
			Public health facilities in Belo Horizonte						Periodontitis was significantly more prevalent among participants with type 2 DM.	
	
									PR – Prevalence of periodontitis/duration of DM:	
									≤ 8 years: Reference	
									> 8 years: 1.63 [1.12–2.38]*	
Gamonal et al. (2010)^21^	Chile	Cross-sectional	1,561 adults (young/senior)	35–44	Yes	Self-reported	Prevalence of moderate to severe CAL, i.e., CAL ≥3, ≥4, ≥5, and ≥ 6 mm present in ≥1 sites.	Age, sex, education, income, smoking	Young adults with DM had significantly higher mean full-mouth CAL.	Diabetic status was not associated with CAL after adjusting for other variables.
			Public health facilities in 15 administrative regions	65–74			Missing teeth, percentage of BoP, mean PD, and mean CAL.			
									**OR - CAL in ≥1 sites:**	
									CAL ≥3 mm: 3.2 [0.7–13.7]	
									CAL ≥4 mm: 1.3 [0.7–2.3]	
									CAL ≥5 mm: 1.3 [0.8–2.0]	
									CAL ≥5 mm: 1.3 [0.9–2.0]	
Haas et al. (2014)^18^	Brazil	Prospective population-based cohort of 5 years of follow-up	653 individuals from the Metropolitan area of Porto Alegre	14	Yes	Self-reported	Percentage of subjects with CAL progression of ≥3mm in ≥2 and ≥4 teeth over 5 years.	Age, gender, marital status, skin color, education, socioeconomic status, interproximal cleaning, dental care, smoking	**RR - CAL progression of ≥3mm in ≥2 teeth:**	DM was significantly associated with CAL progression in the univariable, but not in the multivariate analysis when other variables were considered.
									No diabetes = reference	
									Diabetes: 1.33 [1.07–1.64]*	

									**RR - CAL progression of ≥3mm in ≥4 teeth:**	
									No diabetes = reference	
									Diabetes: 1.52 [1.11–2.09]*	

*Statistically significant. NR: not reported; CAL: clinical attachment loss; PD: probing depth; OR: odds ratio; RRR: relative risk ratio; PR: prevalence ratio; RR: risk ratio; EFP: European Federation of Periodontology; CDC: Center for Disease Control and Prevention; AAP: American Academy of Periodontology; DM: diabetes mellitus.

## Conclusion, research gaps and future perspectives

Compiling scientific epidemiological data on the relationship between periodontitis, diabetes, and smoking in Latin American and Caribbean populations is of utmost importance. This effort can reveal the unique challenges faced in these countries, help guide public health preventive and therapeutic strategies, highlight gaps in the existing literature, and identify areas for future research. Due to the design of the studies, the influence of specific environmental factors and genetic traits in the association between smoking or diabetes and periodontitis could not be determined. Future studies may focus on these areas to help define public policies and recommendations tailored to the Latin American and Caribbean populations.

The findings of this review are consistent with those of a previous systematic review^8^ that underscores cigarette smoking as a risk factor for periodontitis, particularly among heavy smokers. This consistent association across various investigations in different Latin American countries reinforces the harmful impact of smoking on periodontal health, indicating the need for targeted public health interventions. As previously demonstrated,^11^ especially for heavy smokers, the reduction in or cessation of tobacco smoking before periodontal treatment is crucial for the improvement of periodontitis lesions.

There is substantial biological plausibility to support the relationship between diabetes and the onset and progression of periodontitis.^23,24^ Moreover, the assumption that diabetes is a risk factor for periodontitis has been supported by a range of classic and contemporary clinical studies conducted in diverse populations.^25,26^ In this review, while most studies reported associations between diabetes and periodontitis outcomes, unexpectedly, only two found a statistically significant relationship after accounting for confounding factors.^15,22^ However, these findings should be interpreted with caution due to certain methodological aspects of the studies included. These consist of discrepancies in the population ages and methods used to diagnose diabetes, the limited number of prospective studies, and the overall small sample size of participants with diabetes in the studies, which may have reduced the statistical power to observe the real impact of diabetes on periodontitis. Therefore, well-designed population-based prospective studies are still needed for further studies on the association between diabetes and periodontitis in Latin American and Caribbean countries . It is noteworthy that the present review identified only two population-based prospective cohort studies, indicating a gap in longitudinal research within Latin America and the Caribbean. Moreover, the studies were limited to three South American countries (Brazil, Chile, and Colombia), pointing out a need for more extensive research across these regions. Filling these research gaps will offer a more thorough understanding of the association between smoking and diabetes and periodontitis that will be helpful in the development of more effective prevention and treatment approaches in the areas.

Contemporary evidence points out that cessation of exposure to tobacco and diabetes-related lifestyle interventions could significantly reduce the risk of periodontitis.^27-31^ Therefore, oral health professionals should be trained in techniques to open the conversation about the use of tobacco products and awareness of blood glucose levels in their clinical practice.^32^ Considering the proximity of oral health professionals to patients due to multiple visits during the course of their lives, it is important that they feel welcome to discuss smoking-related and diabetes-related themes and that professionals are familiar with the stakeholders themselves and other healthcare professionals who can attend to the patient´s needs. For instance, the Very Brief Advice (VBA) is an intervention that all healthcare professionals can implement in nearly every consultation with smoking patients. It consists of concise, evidence-based recommendations to motivate individuals to quit smoking and directs them to a trained smoking cessation specialist.^33^

Universities, dental associations and government agencies should collaborate to advocate for an agenda of investigations into the determinants of periodontal disease in the Latin America and Caribbean region. Furthermore, there should be a collaborative effort to design and implement strategies to train oral health professionals to discuss smoking and diabetes with their patients and how to handle their questions and needs appropriately. Oral healthcare providers must also be aware of the infrastructure available for patient referrals. The involvement of the oral healthcare team in managing individual risk factors may depend on their ability to collaborate with other professionals, such as psychologists, endocrinologists, nurses, and others. These collaborations can be fostered within the academic setting, with the aim of having professionals who advocate for the comprehensive health of the individual. Policies shall support tobacco cessation programs, screening of patients with pre-diabetes, diabetes and improvement of diabetes care in various healthcare segments. Finally, public health campaigns shall also raise awareness about the risks of smoking and poor diabetes control on oral health. All these measures could potentially contribute to reducing the prevalence and progression of periodontitis in Latin America and Caribbean countries.

## References

[1] Peres MA, Macpherson LM, Weyant RJ, Daly B, Venturelli R, Mathur MR (2019). Oral diseases: a global public health challenge. Lancet.

[2] Watt RG, Daly B, Allison P, Macpherson LM, Venturelli R, Listl S (2019). Ending the neglect of global oral health: time for radical action. Lancet.

[3] Loos BG, Van Dyke TE (2020). The role of inflammation and genetics in periodontal disease. Periodontol 2000.

[4] Genco RJ, Borgnakke WS (2013). Risk factors for periodontal disease. Periodontol 2000.

[5] Martinez-Herrera M, Silvestre-Rangil J, Silvestre FJ (2017). Association between obesity and periodontal disease: a systematic review of epidemiological studies and controlled clinical trials. Med Oral Patol Oral Cir Bucal.

[6] Decker A, Askar H, Tattan M, Taichman R, Wang HL (2020). The assessment of stress, depression, and inflammation as a collective risk factor for periodontal diseases: a systematic review. Clin Oral Investig.

[7] Genco RJ, Borgnakke WS (2020). Diabetes as a potential risk for periodontitis: association studies. Periodontol 2000.

[8] Leite FR, Nascimento GG, Scheutz F, López R (2018). Effect of smoking on periodontitis: a systematic review and meta-regression. Am J Prev Med.

[9] Papapanou PN, Sanz M, Buduneli N, Dietrich T, Feres M, Fine DH (2018). Periodontitis: Consensus report of workgroup 2 of the 2017 World Workshop on the Classification of Periodontal and Peri-Implant Diseases and Conditions. J Periodontol.

[10] Sanz M, Herrera D, Kebschull M, Chapple I, Jepsen S, Beglundh T (2020). EFP Workshop Participants and Methodological Consultants. Treatment of stage I-III periodontitis-The EFP S3 level clinical practice guideline. J Clin Periodontol.

[11] Leite FR, López R, Pajaniaye JB, Nascimento GG (2023). Effect of smoking exposure on nonsurgical periodontal therapy: 1-year follow-up. J Dent Res.

[12] Susin C, Dalla Vecchia CF, Oppermann RV, Haugejorden O, Albandar JM (2004). Periodontal attachment loss in an urban population of Brazilian adults: effect of demographic, behavioral, and environmental risk indicators. J Periodontol.

[13] Susin C, Albandar JM (2005). Aggressive periodontitis in an urban population in southern Brazil. J Periodontol.

[14] Moimaz SA, Zina LG, Saliba O, Garbin CA (2009). Smoking and periodontal disease: clinical evidence for an association. Oral Health Prev Dent.

[15] Silva AM, Vargas AM, Ferreira EF, Abreu MH (2010). Periodontitis in individuals with diabetes treated in the public health system of Belo Horizonte, Brazil. Rev Bras Epidemiol.

[16] Frias AC, Antunes JL, Fratucci MV, Zilbovicius C, Junqueira SR, Souza SF (2011). Population based study on periodontal conditions and socioeconomic determinants in adults in the city of Guarulhos (SP), Brazil, 2006. Rev Bras Epidemiol.

[17] Susin C, Haas AN, Valle PM, Oppermann RV, Albandar JM (2011). Prevalence and risk indicators for chronic periodontitis in adolescents and young adults in south Brazil. J Clin Periodontol.

[18] Haas AN, Wagner MC, Oppermann RV, Rösing CK, Albandar JM, Susin C (2014). Risk factors for the progression of periodontal attachment loss: a 5-year population-based study in South Brazil. J Clin Periodontol.

[19] Schuch HS, Nascimento GG, Peres KG, Mittinty MN, Demarco FF, Correa MB (2019). The controlled direct effect of early-life socioeconomic position on periodontitis in a birth cohort. Am J Epidemiol.

[20] López R, Fernández O, Jara G, Baelum V (2001). Epidemiology of clinical attachment loss in adolescents. J Periodontol.

[21] Gamonal J, Mendoza C, Espinoza I, Muñoz A, Urzúa I, Aranda W (2010). Clinical attachment loss in Chilean adult population: First Chilean National Dental Examination Survey. J Periodontol.

[22] Serrano C, Suarez E (2019). Prevalence of severe periodontitis in a Colombian adult population. J Int Acad Periodontol.

[23] Graves DT, Ding Z, Yang Y (2020). The impact of diabetes on periodontal diseases. Periodontol 2000.

[24] Bitencourt FV, Nascimento GG, Costa SA, Andersen A, Sandbæk A, Leite FR (2023). Co-occurrence of periodontitis and diabetes-related complications. J Dent Res.

[25] Chávarry NG, Vettore MV, Sansone C, Sheiham A (2009). The relationship between diabetes mellitus and destructive periodontal disease: a meta-analysis. Oral Health Prev Dent.

[26] Nascimento GG, Leite FR, Vestergaard P, Scheutz F, López R (2018). Does diabetes increase the risk of periodontitis? A systematic review and meta-regression analysis of longitudinal prospective studies. Acta Diabetol.

[27] Rosa EF, Corraini P, Inoue G, Gomes EF, Guglielmetti MR, Sanda SR (2014). Effect of smoking cessation on non-surgical periodontal therapy: results after 24 months. J Clin Periodontol.

[28] Leite FR, Nascimento GG, Baake S, Pedersen LD, Scheutz F, López R (2019). Impact of smoking cessation on periodontitis: a systematic review and meta-analysis of prospective longitudinal observational and interventional studies. Nicotine Tob Res.

[29] Saengtipbovorn S, Taneepanichskul S (2015). Effectiveness of lifestyle change plus dental care program in improving glycemic and periodontal status in aging patients with diabetes: a cluster, randomized, controlled trial. J Periodontol.

[30] Nishihara U, Tanabe N, Nakamura T, Okada Y, Nishida T, Akihara S (2017). A periodontal disease care program for patients with type 2 diabetes: A randomized controlled trial. J Gen Fam Med.

[31] Mizutani K, Minami I, Mikami R, Kido D, Takeda K, Nakagawa K (2024). Improvement of periodontal parameters following intensive diabetes care and supragingival dental prophylaxis in patients with type 2 diabetes: a prospective cohort study. J Clin Periodontol.

[32] Ramseier CA, Woelber JP, Kitzmann J, Detzen L, Carra MC, Bouchard P (2020). Impact of risk factor control interventions for smoking cessation and promotion of healthy lifestyles in patients with periodontitis: a systematic review. J Clin Periodontol.

[33] Papadakis S, McEwen A (2021). Very brief advice on smoking PLUS (VBA+).

